# Recent Advances in the 5q- Syndrome

**DOI:** 10.4084/MJHID.2015.037

**Published:** 2015-05-20

**Authors:** Andrea Pellagatti, Jacqueline Boultwood

**Affiliations:** Leukaemia & Lymphoma Research Molecular Haematology Unit, Nuffield Division of Clinical Laboratory Sciences, Radcliffe Department of Medicine, University of Oxford, and BRC Blood Theme, NIHR Oxford Biomedical Centre, Oxford University Hospitals, Oxford, United Kingdom

## Abstract

The 5q- syndrome is the most distinct of the myelodysplastic syndromes (MDS) and patients with this disorder have a deletion of chromosome 5q [del(5q)] as the sole karyotypic abnormality. Several genes mapping to the commonly deleted region of the 5q- syndrome have been implicated in disease pathogenesis in recent years. Haploinsufficiency of the ribosomal gene *RPS14* has been shown to cause the erythroid defect in the 5q- syndrome. Loss of the microRNA genes miR-145 and miR-146a has been associated with the thrombocytosis observed in 5q- syndrome patients. Haploinsufficiency of *CSNK1A1* leads to hematopoietic stem cell expansion in mice and may play a role in the initial clonal expansion in patients with 5q- syndrome. Moreover, a subset of patients harbor mutation of the remaining *CSNK1A1* allele. Mouse models of the 5q- syndrome, which recapitulate the key features of the human disease, indicate that a p53-dependent mechanism underlies the pathophysiology of this disorder. Importantly, activation of p53 has been demonstrated in the human 5q- syndrome. Recurrent *TP53* mutations have been associated with an increased risk of disease evolution and with decreased response to the drug lenalidomide in del(5q) MDS patients. Potential new therapeutic agents for del(5q) MDS include the translation enhancer L-leucine.

## The 5q- syndrome: candidate genes and pathophysiology

The myelodysplastic syndromes (MDS) are heterogeneous clonal hematopoietic stem cell (HSC) malignancies characterized by ineffective hematopoiesis, peripheral blood cytopenias, and typically patients have a hypercellular bone marrow. MDS patients frequently show disease progression (approximately 40% of cases) to acute myeloid leukemia (AML).^1^

Chromosomal monosomies and deletions are commonly observed in MDS. Cytogenetic abnormalities are present in approximately 50% of *de novo* MDS and 80% of therapy-related MDS cases.^2^ Interstitial deletion within the long arm of chromosome 5 [del(5q)] is one of the most common karyotypic abnormalities reported in *de novo* MDS, occurring in approximately 10–20% of patients with this disorder.^2^

Patients are defined as 5q- syndrome when they have a del(5q) as the sole karyotypic abnormality and a medullary blast count of less than 5%.^3^,^4^ The 5q- syndrome was first described by Van den Berghe et al in 1974^5^ and is the most distinct of the MDS with a clear genotype-phenotype relationship. Patients with the 5q- syndrome show macrocytic anemia, hypolobulated megakaryocytes, a normal or high platelet count and a good prognosis with approximately 10% of patients transforming to AML.^6^,^7^

The del(5q) is considered to mark the location of one or more genes the loss of which may affect important processes involved in normal hematopoiesis.^8^ The commonly deleted region (CDR) of the 5q- syndrome was identified over 20 years ago using molecular mapping and fluorescent in situ hybridization techniques by Boultwood et al^9^ and was progressively narrowed to a ~1.5Mb interval at 5q32-q33 flanked by the DNA marker D5S413 and the *GLRA1* gene.^10^,^11^ Genomic annotation of the CDR of the 5q- syndrome highlighted several promising candidate genes mapping to the CDR, including the tumor suppressor gene *SPARC*, the ribosomal protein gene *RPS14* and several microRNA genes.^10^,^12^ Mutation screening of all 40 genes within the CDR was performed in ten 5q- syndrome patients using Sanger sequencing several years ago and no mutations were identified.^12^ The absence of mutations in genes in the CDR was suggestive of haploinsufficiency (a dosage effect resulting from the loss of one allele of a gene)^13^ being an important mechanism in the 5q- syndrome.

In a study published in 2007, the transcriptome of bone marrow CD34^+^ cells was investigated in a cohort of ten patients with the 5q- syndrome using microarray-based gene expression profiling.^12^ Several candidate genes mapping to the CDR of the 5q- syndrome showed haploinsufficiency in 5q- syndrome patients, including *RPS14*, encoding a component of the 40S ribosomal subunit, and *CSNK1A1*, encoding a serine/threonine kinase.^12^ Crucially, these two genes would be shown in subsequent studies^14^,^15^ to have an important role in the molecular pathogenesis of the 5q- syndrome.

In a landmark study by Ebert et al in 2008, *RPS14* was identified as a 5q- syndrome gene using a RNA-mediated interference (RNAi)-based screen of each gene within the CDR.^14^ Knockdown of RPS14 to haploinsufficient levels in normal HSC resulted in a block in erythroid differentiation with relative preservation of megakaryocytic differentiation. Forced expression of *RPS14* in primary bone marrow cells from 5q- syndrome patients rescued the phenotype, demonstrating the important role of RPS14 in the 5q- syndrome.^14^ In addition, RPS14 haploinsufficiency resulted in a block in the processing of pre-ribosomal RNA and in abrogation of 40S ribosomal subunit formation.^14^ Studies by Pellagatti et al have shown that CD34^+^ cells from patients with the 5q- syndrome have defective expression of many ribosomal- and translation-related genes.^16^,^17^ The results of these studies suggest that the 5q- syndrome is a disorder of aberrant ribosome biogenesis, and the 5q- syndrome is now considered to be a ribosomopathy.^18^ There is a strong analogy between the 5q- syndrome and Diamond-Blackfan anemia (DBA),^12^ a disorder which is similarly caused by haploinsufficiency of ribosomal protein genes, including *RPS19*.^19^

A mouse model of the 5q- syndrome has been generated by Barlow et al using large-scale chromosomal engineering.^20^ Mice with haploinsufficiency of the *Cd74-Nid67* interval (which is syntenic to the CDR of the human 5q- syndrome and includes *Rps14*) recapitulated the key features of the human disease, including a macrocytic anemia and monolobulated megakaryocytes in the bone marrow.^20^ This ‘5q- mouse’ showed defective bone marrow progenitor development and an accumulation of p53 protein with increased apoptosis was observed in the bone marrow cells, similar to that observed in animal models of DBA.^21^ The progenitor cell defect could be rescued by intercrossing the ‘5q- mouse’ with p53-deficient mice, providing the first evidence that a p53-dependent mechanism underlies the pathophysiology of the 5q- syndrome.^20^

Recently, a murine model for conditional, heterozygous inactivation of *Rps14* in the bone marrow has been generated.^22^ *Rps14* haploinsufficient mice showed significantly reduced hemoglobin and red blood cell counts with a significantly higher MCV. Bone marrow analysis confirmed an erythroid differentiation defect, with a significant increase in hypolobulated megakaryocytes. *Rps14* haploinsufficient mice also showed reduced protein synthesis and p53 induction in late-stage erythroblasts. Genetic inactivation of p53 rescued the erythroid phenotype: the erythroid differentiation defect was restored in *Rps14*^−/+^*p53*^−/+^ mice.^22^ This murine model shows that haploinsufficiency of *Rps14* is sufficient to recapitulate the erythroid and megakaryocytic phenotype observed in the 5q- syndrome.

Importantly, induction of p53 and up-regulation of the p53 pathway has been shown to occur in the human 5q- syndrome.^23^ Immunohistochemical analysis showed moderate to strong p53 expression in erythroid cells in bone marrow trephine sections from patients with the 5q- syndrome, and gene expression profiling demonstrated that the p53 pathway is significantly deregulated in the CD34^+^ cell population of these patients.^23^ The accumulation of p53 protein in bone marrow erythroid precursors of patients with the 5q- syndrome has been confirmed in other studies.^24^,^25^

Activation of p53 has been shown to occur selectively in erythroid cells differentiated from human HSC with shRNA-based knockdown of *RPS14*.^24^ Induction of p53 resulted in erythroid-specific accumulation of p21, cell cycle arrest and apoptosis, consistent with the failure of erythropoiesis observed in the 5q- syndrome.^24^ Inhibition of p53 using pifithrin-α in culture rescued the erythroid defect, suggesting that p53 activation may represent a therapeutic target in MDS with del(5q).^24^

Thus several converging lines of evidence^20^,^21^,^23^,^24^ demonstrate that ribosomal stress leads to activation of the p53 pathway, a key effector of erythroid hypoplasia in both del(5q) MDS and congenital ribosomopathies.

### CSNK1A1

A recent study by Schneider et al has shown that *CSNK1A1* plays a central role in the pathogenesis of del(5q) MDS.^15^ *CSNK1A1* encodes CK1α, a central regulator of β-catenin^26^ which is a major driver of stem cell self-renewal.^15^ Heterozygous inactivation of *Csnk1a1* in mice led to β-catenin activation and expansion of HSCs,^15^ suggesting that *CSNK1A1* haploinsufficiency may be the mechanism underlying the initial clonal expansion in patients with the 5q- syndrome. Mutations of *CSNK1A1* were identified in approximately 7% of MDS del(5q) cases analyzed.^15^ A *CSNK1A1* mutation was reported in a del(5q) MDS patient in a previous study.^27^ Interestingly, Schneider et al showed that expression of mutant *CSNK1A1* resulted in β-catenin activation and HSC cell cycle progression. Thus *CSNK1A1* mutations are recurrent in a small proportion of del(5q) MDS patients, and there is evidence that these mutations may drive clonal dominance. Furthermore, Csnk1a1 haploinsufficiency was shown to sensitize cells to casein kinase inhibition, indicating that CSNK1A1 is a potential new therapeutic target for the treatment of del(5q) MDS.^15^

### MicroRNA genes

It has been suggested that haploinsufficiency of miR-145 and miR-146a, two miRNA genes that map within and adjacent to the CDR respectively,^12^ may be the cause of other key features of the 5q- syndrome, namely hypolobulated megakaryocytes and thrombocytosis. The study by Starczynowski et al showed down-regulation of miR-145 and miR-146a in the CD34^+^ cells of patients with the 5q- syndrome.^28^ Knockdown of these miRNAs in mouse HSCs resulted in thrombocytosis, mild neutropenia and megakaryocytic dysplasia.^28^ Kumar et al have identified the *FLI1* gene, encoding a transcription factor involved in megakaryopoiesis, as a critical target of miR-145 and have shown that patients with del(5q) MDS have increased expression of *FLI1*.^29^ Inhibition of miR-145 or overexpression of *Fli-1* resulted in an increase in the production of megakaryocytic cells relative to erythroid cells.^29^ These data suggest that deficiency of miR-145 and miR-146a may underlie the thrombocytosis observed in some 5q- syndrome patients.

## Cell of Origin

The 5q- syndrome is a disorder originating in the human HSC. Using immunophenotyping and FISH, Jaju et al showed B-cell involvement in one of three cases with the 5q- syndrome.^30^ In the study of Nilsson et al, no T-cell involvement was observed in nine patients with del(5q), but one patient had B-cell involvement.^31^ A minimum of 94% of cells in the minor CD34^+^CD38^−^ HSC compartment carried a del(5q) in all patients analyzed and 5q aberrations were found in 25–90% of purified CD34^+^CD19^+^ pro-B cells in three of five patients.^31^ These data strongly suggest that 5q deletions occur in HSCs with a combined lympho-myeloid potential and that 5q deletions represent an early event in MDS pathogenesis.^31^ A subsequent gene expression profiling study of highly purified 5q-deleted CD34^+^CD38 Thy1^+^ cells in 5q- MDS identified a molecular signature supporting a HSC origin for this disorder.^32^

In a recent study, Woll et al elegantly demonstrated that the MDS are propagated by rare and distinct human cancer stem cells in vivo.^27^ A total of 34 somatic lesions, including del(5q) and driver mutations, were identified in bulk bone marrow cells of 15 patient with lower-risk MDS and all these lesions could be tracked back to the stem cell compartment. In MDS cases with del(5q) and additional driver mutations, acquisition of del(5q) preceded recurrent gene mutations, with the exception of four MDS cases with sideroblastic anemia in which the del(5q) was preceded by *SF3B1* gene mutations. In all cases with isolated del(5q) or RAEB1/RCMD, the del(5q) was predicted to be the first (or only) genetic lesion. These data are compatible with the del(5q) being the initiating and potentially the only genetic lesion required for the development of MDS with isolated del(5q).

## Treatment

The immunomodulatory drug lenalidomide has been shown to have dramatic therapeutic efficacy in patients with the 5q- syndrome and other MDS patients with del(5q).^33^,^34^ A large multicentre phase II trial by List et al evaluated lenalidomide treatment response in 148 MDS patients with del(5q): transfusion independency and a complete cytogenetic remission was achieved in 67% and 45% of patients respectively.^33^ Lenalidomide is now considered the standard of care for the treatment of transfusion dependent anemia in lower risk del(5q) MDS patients.^35^

Lenalidomide has been shown to inhibit the growth of MDS del(5q) erythroblasts but did not affect normal cells in culture.^36^ The mode of action of lenalidomide has been investigated in several studies. Lenalidomide has been shown to upregulate several genes, including the tumor suppressor gene *SPARC* and the TGF-β family member activin A, in hematopoietic progenitor cells from patients with del(5q) MDS and normal individuals.^36^,^37^ SPARC, located at 5q32-q33 within the CDR of the 5q- syndrome, has anti-proliferative, anti-adhesive, and anti-angiogenic properties, all known effects of immunomodulatory drugs.^38^ Wei et al^39^ demonstrated that lenalidomide inhibits two phosphatases, Cdc25C and PP2Acα. The genes for these phosphatases are located on 5q and are deleted in most patients with del(5q) MDS. Cdc25C and PP2Acα are co-regulators of the G2-M checkpoint in the cell cycle and thus their inhibition by lenalidomide leads to G2 arrest and apoptosis. These data suggest that haploinsufficiency of Cdc25C and PP2Acα in del(5q) MDS causes an enhanced sensitivity to lenalidomide.^39^

Lenalidomide has been shown to promote degradation of p53 by inhibiting auto-ubiquitination of MDM2 in del(5q) MDS.^25^ It has been suggested that lenalidomide restores MDM2 functionality in the 5q-syndrome to overcome p53 activation in response to ribosomal stress.^25^ A recent study reported that lenalidomide induces the ubiquitination and consequent degradation of *CSNK1A1* by the CRBN-CRL4 E3 ubiquitin ligase.^40^ Knockdown of *CSNK1A1* sensitized primary CD34^+^ cells to lenalidomide, suggesting that haploinsufficiency of *CSNK1A1* might increase lenalidomide sensitivity in del(5q) hematopoietic cells.^40^

These data implicate several genes mapping to del(5q) in the mode of action of lenalidomide in del(5q) MDS.

It would be valuable to identify predictive factors for response to lenalidomide, and an erythroid differentiation signature that predicts response to lenalidomide in MDS has been identified.^41^ A correlation of clinical response and response duration with induction of the microRNA miR-145 by lenalidomide in CD34^+^ cells from patients with MDS and the del(5q) has been recently proposed.^37^ Importantly, the presence of *TP53* mutation has been shown to influence negatively the response to lenalidomide in del(5q) MDS in several studies. In the study by Jädersten et al the probability of a complete cytogenetic response to lenalidomide was significantly lower in *TP53* mutated patients.^42^ Another study confirmed the importance of *TP53* mutational status for response to lenalidomide treatment: wild-type *TP53* status showed a tendency for hematological response, while none of the cases with mutated *TP53* achieved a complete cytogenetic response.^43^ In a recent study by Saft et al, immunohistochemical analysis of p53 was performed in bone marrow biopsies from 85 lower-risk del(5q) MDS patients treated with lenalidomide.^44^ Strong p53 expression in ≥1% of bone marrow progenitor cells was observed in 35% of patients and was significantly associated with shorter survival, higher risk of evolution to AML and a lower cytogenetic response rate to lenalidomide.

**Figure 1:**
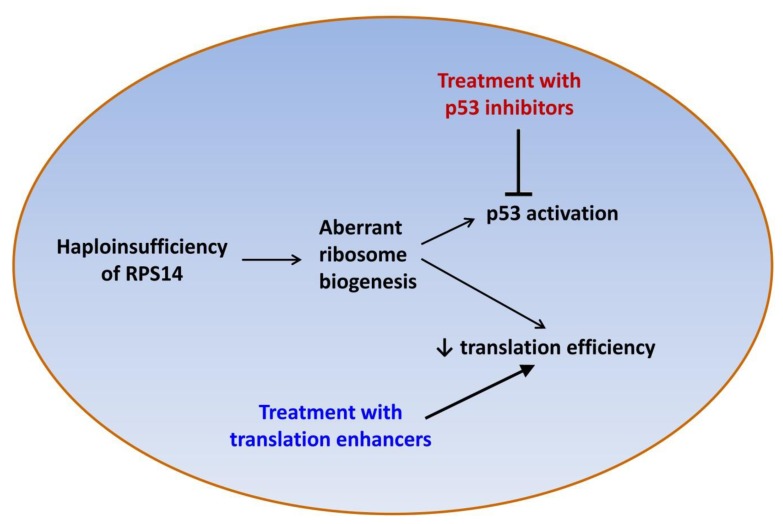
Mechanisms underlying the erythroid defect in the 5q- syndrome and potential new therapeutic targets.

Lenalidomide is clearly an effective treatment for lower-risk, transfusion-dependent MDS patients with del(5q), however not all patients respond to lenalidomide and approximately half of MDS patients with del(5q) acquire resistance to the drug within two to three years.^33^ There is thus a clinical need for novel treatments for MDS patients with del(5q). Potential new therapeutic agents for this group of patients include the translation enhancer L-leucine^45^,^46^ and the p53 inhibitor cenersen^47^ (Figure 1).

### L-leucine

The HSCs of patients with the 5q- syndrome show defective ribosome biogenesis^14^ and deregulation of many ribosomal- and translation-related genes.^17^ Defective ribosome biogenesis may result in a reduction in the efficiency of mRNA translation and total protein production has been shown to be significantly decreased in erythroid cells with knockdown of *RPS14*.^46^ This defect in mRNA translation represents a potential therapeutic target and there is evidence suggesting that the translation enhancer L-leucine may have some efficacy in ribosomopathies. Pospisilova et al described a DBA patient who showed a marked improvement in anemia and became transfusion-independent after treatment with L-leucine.^48^ Yip et al reported that L-leucine treatment of cultured erythroblasts derived from CD34^+^ cells of healthy controls with RPS14 knockdown and from CD34^+^ cells of del(5q) patients resulted in an increase in proliferation, erythroid differentiation and mRNA translation.^46^ Zebrafish models of del(5q) MDS and DBA treated with L-leucine showed increased hemoglobinization and red cell numbers and reduced developmental defects.^45^ Similarly, L-leucine treatment of a mouse model of DBA resulted in improved hemoglobin concentration and in an increase in the number of erythrocytes.^49^ There is evidence suggesting that the enhanced erythroid progenitor cell growth and differentiation observed in animal and cellular models of the 5q- syndrome and DBA treated with L-leucine occurs through activation of the mTOR pathway.^45^,^50^ These data support the rationale for clinical trials of L-leucine as a therapeutic agent for the 5q- syndrome and DBA.

A recent report described three MDS patients with isolated del(5q) who were treated with L-leucine for up to three months. No adverse effects were observed during L-leucine treatment, however none of the patients showed an improvement in the cytopenia or transfusion requirements.^51^ Leucine absorption tests may be useful in determining the optimal dose^48^ and in vitro measurement of basal and post-L-leucine translation levels could help identifying patients that are more likely to respond to L-leucine therapy.^48^ It will be important to determine the efficacy of L-leucine treatment in larger patient cohorts within the context of clinical trials. Indeed, clinical trials evaluating the therapeutic use of L-leucine in DBA are underway in the US and in Russia (NCT02386267 and NCT01362595, www.clinicaltrials.gov).

### Cenersen

Recently, cenersen, a clinically active 20-mer antisense oligonucleotide complementary to exon 10 of *TP53*, has been shown to suppress p53 expression and restore erythropoiesis in del(5q) MDS patient cells in culture.^47^ Cenersen treatment of RPS14-deficient erythroblasts significantly reduced cellular p53 and PUMA expression, decreased apoptosis and increased cell proliferation. Cenersen significantly suppressed nuclear p53 in CD34^+^ cells isolated from del(5q) MDS patients. Erythroid burst recovery increased in proportion to the magnitude of p53 suppression without del(5q) clonal suppression. Dexamethasone, a p53 antagonist, was added to lenalidomide treatment in eight lower-risk, transfusion-dependent, del(5q) MDS patients with acquired drug resistance. Transfusion independence was restored in five patients, with expansion of erythroid precursors and decreased p53 expression. This study shows that targeted suppression of p53 restores effective erythropoiesis in lenalidomide-resistant del(5q) MDS.^47^ A clinical trial testing the benefits of cenersen in lower-risk MDS patients is in progress (NCT02243124, www.clinicaltrials.gov). Data from AML and CLL clinical trials have shown increased cytotoxicity and enhanced sensitivity to conventional chemotherapy when given in combination with cenersen, with no safety issues associated with the use of cenersen.^52^,^53^ Transient pharmacological inhibition of p53 has been shown not to increase the incidence of cancer in a murine carcinogenicity model.^54^ Suppression of p53 could possibly be a therapeutic option in humans if the tumor suppressor function of p53 is only transiently abrogated and no long-term adverse effects are observed.

## Disease Progression

Approximately 10% of patients with 5q- syndrome progress to AML and the underlying genetic events driving disease evolution are poorly understood.^6^ Some studies have shed light on the molecular basis of leukemic transformation in del(5q) MDS.

There is evidence to suggest that mutation of *TP53*, resulting in the inactivation of the p53 protein, may be one of the molecular events involved in the clonal progression of the 5q- syndrome to AML.^42^,^55^–^57^ Using deep-sequencing technology, Jädersten et al have demonstrated that small hematopoietic cell subclones with *TP53* mutation could be detected at an early disease stage in 18% of patients with MDS with del(5q). The *TP53* mutations were present years before disease progression and were associated with an increased risk of evolution to AML.^42^ These findings indicate the existing heterogeneity even within an MDS subtype harboring a single cytogenetic abnormality.

Fernandez-Mercado et al have used a next-generation sequencing-based panel, targeting 25 genes mutated in various myeloid malignancies, to study a cohort of 43 early and advanced MDS cases with del(5q).^55^ Overall, 45% of early and 67% of advanced MDS cases presented at least one mutation. *TP53* and *ASXL1* were the genes with the highest mutation frequency (25% of patients for each gene) among advanced cases, and showed a lower mutation frequency in cases of 5q- syndrome (4.5% and 13.6%, respectively), suggesting a role in disease progression in del(5q) MDS.

A recent study using whole exome sequencing on paired samples from two MDS cases with del(5q) and one MDS case without del(5q) before and after progression to AML, showed that most mutations identified in the two del(5q) cases were present at the AML stage only.^58^ In contrast, most mutations identified in the case without del(5q) were present at both the MDS and the AML stage. This study identified recurrent mutations of *TP53* and *RYR1* at the AML stage of the two del(5q) patients analyzed.^58^ The *RYR1* gene encodes a calcium release channel and may represent a new recurrently mutated gene associated with MDS del(5q) transformation to AML. Interestingly, 67% of infant leukemia patients with AML were shown to be compound heterozygotes for *RYR1* and *FLG*.^59^

It is well recognized that mutated p53 can lead to genetic instability and disease progression in cancer and leukemia. Mounting evidence suggests that p53 plays an important role in the development and progression of the 5q- syndrome, with activation of wild-type p53 resulting in increased apoptosis and defective erythropoiesis in the early stages of the disease, followed by an expansion of a small subclone harboring mutant (inactivated) p53 in some patients as the disease progresses, leading to leukemic transformation. It may be speculated that if increased p53 activity inhibits the growth of primitive del(5q) malignant cells, then there could be a selective pressure for these cells to mutate or lose the *TP53* gene. In support of this suggestion, the del(5q) and *TP53* mutations do appear to strongly associate, indicating a likely synergy between these abnormalities.

It has been shown that, while *NPM1* deletion is an uncommon event in patients with the 5q- syndrome, it occurs in 40% of cases with high-risk MDS/AML with complex karyotypes and 5q deletion and is therefore associated with more advanced forms of del(5q) MDS.^60^

## Conclusions

Several cooperating events seem to be necessary in the development of the 5q- syndrome. The studies described above show that p53 activation secondary to RPS14 haploinsufficiency underlies the anemia observed in patients with the 5q- syndrome. Loss of the miRNA genes miR-145 and miR146a seems to play a role in the development of the megakaryocytic abnormalities observed in this disorder. The molecular abnormality that confers a clonal growth advantage in the 5q- syndrome has remained elusive for a long time, but it has now been shown that haploinsufficiency of CSNK1A1 may be the cause of the initial clonal expansion in 5q- syndrome patients. Thus molecular abnormalities associated with the major features of the 5q- syndrome have now been identified in a number of genes that map to the CDR.

Mutations of *TP53* have been associated with disease progression in del(5q) MDS. Other gene mutations have been described in a small number of del(5q) patients that evolved to AML and their potential role in disease transformation will need to be confirmed in larger patient cohorts. It is possible that there are other events still unidentified that may be important in disease pathogenesis. Whole genome sequencing studies may identify new changes in intronic or regulatory regions of genes mapping within the CDR of the 5q- syndrome or elsewhere in the genome that are associated with the development and/or progression of this disorder. Haploinsufficiency of other genes may also be involved in disease pathogenesis.

**Figure 2:**
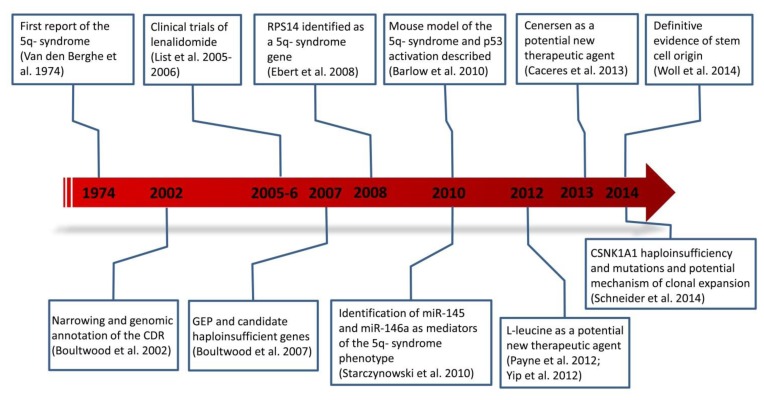
Timeline and milestones in the study of the 5q- syndrome.

Great progress has been made over the past decade in the elucidation of the molecular basis of the 5q- syndrome (Figure 2), and new insights into disease mechanisms are leading to the development of novel treatments for the 5q- syndrome.
